# Collecting data for sexually transmitted infections (STI) surveillance: what do patients prefer in Flanders?

**DOI:** 10.1186/1472-6963-7-149

**Published:** 2007-09-20

**Authors:** Veronique Verhoeven, Annelies Colliers, Ann Verster, Dirk Avonts, Lieve Peremans, Paul Van Royen

**Affiliations:** 1 Centre for General Practice, University of Antwerp, Universiteitsplein 1, 2610 Wilrijk, Belgium

## Abstract

**Background:**

STI surveillance systems are subject to qualitative and quantitative underreporting. General practitioners (GPs), who are key subjects in case reporting, explain their underreporting partly by their observation that taking a sexual history is embarrassing for patients, and that patients are reluctant to disclose information on their sexual practices. In this study we examine patients' willingness to provide data for STI surveillance.

**Methods:**

A questionnaire-based survey in a stratified population sample of 300 patients aged 18–60 years.

**Results:**

The large majority of respondents stated to be willing to give information on their sexual practices for the purpose of STI surveillance. They preferred to answer sexual history questions to their GP; filling in a form on the internet was the second best option.

**Conclusion:**

Based on these results, it is unlikely that the cooperation of patients would be a weak link in STI surveillance strategies. This observation, together with the fact that the majority of patients at risk for STIs have regular access to general practice services, justify renewed efforts to enliven primary care-based STI surveillance strategies.

## Background

Surveillance data are essential resources for understanding and controlling the increasing incidence of STIs in Europe [1].

Belgium [2], as most other European countries [1], has several complementary STI surveillance systems in operation. Case reporting is mandatory for syphilis, gonorrhoea, hepatitis B/C and scabies. Furthermore, a voluntary sentinel laboratory system and a recently introduced sentinel case reporting system collect information on a larger number of STIs.

The resulting databases are managed by different authorities and all systems contend with underreporting of cases as well as incomplete provision of data on case reports. Although laboratory data show that a substantial number of STI cases are detected by GPs, coverage of case reporting in primary care is very low.

An earlier focus group study in general practice [3] unveiled that GPs have many barriers towards STI surveillance. In that study GPs were worried about embarrassing patients by asking sensitive questions, and they perceived patients to be reluctant to disclose information on their sexual lives to their doctors. This seems to be an important reason for GPs not to participate in STI surveillance.

Patients' attitude towards data collection obviously is vital for the success of any surveillance system. However, it has not been examined yet whether the arguments of GPs about reluctance of patients are justified; therefore in this study we surveyed to what extent a sample of potential patients would be willing to participate in providing data for STI surveillance in case they would be diagnosed with an STI. We also asked respondents how they felt this data collection should be organised.

## Methods

A survey was carried out in July 2006 in Antwerp, Belgium, in a population sample of 300 people, using a written, anonymous, structured questionnaire [see additional file 1]. The sample was stratified according to the gender and age distribution of the population in Flanders. Two medical students distributed questionnaire forms to visitors of a shopping mall who were resting on benches in the shadow of Antwerp cathedral. Coloured questionnaire forms were used for different age- and gender groups. Eligible participants were aged 18–60 years; we doubled the proportion of people under 30 in the sample as they are considered more at risk for STIs. People who were under 18 or over 60, who did not live in Flanders or who did not speak Dutch were considered as non-eligible, and were not counted in calculations of the response rate. The (relatively few) people who declined to talk to the recruiters or who chose not to participate were, as non-respondents, included in the denominator. Respondents filled in their questionnaire on the spot; after returning it in a sealed envelope they received a free film ticket as an incentive.

The study protocol was approved by the Medical Ethics Committee of Antwerp University. The fact that respondents agreed to fill in the questionnaire was considered as informed consent.

Descriptive and univariate statistics were performed using SPSS for Windows version 13.0. Chi square statistics, t-test or Mann-Whitney U-test were applied, depending on the variables' distribution.

## Results

Three hundred forms were returned; the response rate was 90%. Mean age of participants was 32.73 years (range 18–68); 48% were female and 91% were born in Belgium. Thirty-eight per cent of participants had a secondary school education or less; 62% had received higher education. Of all participants, 92% had a regular GP, 92% used the internet, 88% had been sexually active in the past year, 12% had had more than 1 sex partner in the past year, and 11% were homosexual or bisexual. An STI had ever been diagnosed in 6%, and 39% had ever been tested for STIs.

The vast majority of respondents found it acceptable that their GP would ask them sexual history questions in the context of STI surveillance. Filling in a form on the internet was the second best option. (table 1). Questions on contacts with a prostitute were the least acceptable.

**Table 1:** Number (%) of respondents who would answer sexual history questions to various authorities

"Below, you find the questions that you would be asked, if you would be diagnosed with an STI. **Who would you allow to ask the following questions?"**					
	My GP	Someone else (on the phone)	Someone else (by mail)	I answer the question on an internet form	No one is allowed to ask me this question

Which symptoms do you have?	295 (98)	24 (8)	52 (17)	85 (28)	0 (0)
Did you have an STI before?	274 (91)	35 (12)	59 (20)	90 (30)	9 (3)
Do you have an idea who might have passed the infection on to you?	267 (89)	27 (9)	44 (15)	69 (23)	15 (5)
Do you have a regular sex partner?	275 (92)	46 (15)	65 (22)	88 (29)	8 (3)
Do you have occasional sex partners?	255 (85)	34 (12)	55 (18)	80 (27)	25 (8)
When might you have gotten the infection?	281 (94)	29 (10)	56 (19)	85 (28)	4 (1)
Where (in which town) might you have gotten the infection?	250 (83)	29 (10)	56 (19)	82 (27)	26 (9)
Did you visit a prostitute?	220 (73)	19 (6)	39 (13)	69 (23)	59 (20)

The willingness to disclose information (which we defined as "the number of suggested options which were considered as acceptable", which means in this study almost the same as "whether other options than the GP were acceptable") was independent of respondents' gender, ever having had a test for STIs, and number of partners in the past year. Willingness was higher in respondents under 40 (p = 0.005), in homosexuals and bisexuals (p = 0.008), and in people who had ever had an STI (p = 0.039). Homosexuals/bisexuals had no higher incidence of STIs than heterosexuals in this sample (p = 0.1). Respondents who had a regular GP were more willing to answer questions to their GP than respondents who had no regular GP. Forty-three per cent of female respondents had a female regular GP, versus 23% of male respondents (p = 0.002). The gender of the GP was not a significant determinant for the willingness to disclose information.

## Discussion

This survey shows that the collection of data for STI surveillance is acceptable for a sample of potential patients, many of whom have already, at some point in their lives, felt the necessity to have a test for STIs. Respondents report to be willing to answer even sensitive sexual history questions, and they prefer face-to-face contact with a GP above more impersonal options such as filling in an internet form or being contacted by mail or telephone. These findings are in contradiction with GPs' scepticism regarding the feasibility of sexual history taking in primary care [3,4].

For Flanders or Belgium, no reliable data are available on the importance of the general practice setting in STI diagnoses; however, it is well known that a large part of the population at risk has access to general practice medicine – and to this setting only.

This survey has a number of limitations; first, other reasons why GPs do not participate in STI surveillance, such as lack of time availability for interviewing patients and filling out STI reported forms are not addressed in this study.

Second, although the free cinema ticket and the relaxed athmosphere in which the students asked people to participate led to a surprisingly high response rate of 90%, some participation bias (involuntary selection of participants by the students) cannot be excluded. It is possible that a sample of shopping mall visitors is not fully representative for the general population, however, the high response rate, the strong direction in which the results point and the lack of other research on this subject make our study an interesting starting point. The sample was representative for the Flemish population with regard to age and gender distribution. We deliberately chose not to recruit participants in another setting (for example GPs' waiting rooms) to avoid bias in favour of GPs. Third, this study measures patients' intention to provide information, which may possible not be an accurate reflection of the actual willingness to provide this information at the moment of consultation.

Case reporting in general practice offers the opportunity not only to record demographical data on STI cases, but also to take a detailed personal and sexual history, and thus gather valuable information on sexual history and sexual practices. With this information, surveillance data can be used not only to determine incidence trends, but also to unveil transmission routes, identify risky behaviours and define target groups to focus interventions[5]. These observations, together with the fact that the majority of patients at risk for STIs have regular access to general practice services, justify renewed efforts to enliven primary care-based STI surveillance strategies.

## Competing interests

The author(s) declare that they have no competing interests.

## Authors' contributions

VV conceived of the study, performed statistical analysis and drafted the manuscript. AC and AV collected data, coordinated the study and assisted in writing the manuscript. LP, DA and PVR were involved in study design, participated in discussing the results and revised the manuscript. All authors have read and approved the final manuscript.

## Pre-publication history

The pre-publication history for this paper can be accessed here:



## Supplementary Material

Additional file 1 Questionnaire. The structured questionnaire which was used in the study. Click here for file
