# Supplementary material for: Collecting data for sexually transmitted infections (STI) surveillance: what do patients prefer in Flanders?
Source: BMC Health Serv Res. 2007 Sep 20;7:149. doi: 10.1186/1472-6963-7-149 (PMC2039737; doi:10.1186/1472-6963-7-149)
Supplement: Additional file 1 — Questionnaire. The structured questionnaire which was used in the study. [file 1472-6963-7-149-S1.pdf]

Universiteit Antwerpen

Questionnaire on the reporting of STIs

In the past years, sexually transmitted infections (STIs) were diagnosed more frequently than before in Flanders. STIs are for example syphilis, gonorrhea, chlamydia or HIV, which are contagious and are transmitted through sexual contact.
If such an infection is diagnosed, for example in a patient who visits his/her GP, a number of data need to be registered. These data are used to examine how these infections spread, and how further transmission can be prevented.

The government would like to know how patients, like you, think about registratrion of information on STIs.

In this questionnaire we ask for your opinion; there are no wrong answers! This is an anonymous questionnaire, you don't need to write your name on it.

Part one: how should information be collected?

Suppose that your GP would diagnose you with a sexually transmitted infection.
Who would you allow to ask you the following questions, for the purpose of STI surveilance?
For every question, four possible answers are suggested below. Please consider which one(s) is (are) acceptable for you.

(1) **My GP** can ask me this question
(2) **Someone else, for example an official** who is specially appointed for this purpose (for example a health inspector) can ask me this question **over the telephone** (my GP passes my phone number on to this official)
(3) **Someone else, for example an official** who is specially appointed for this purpose can ask me this question **by sending me a letter** (my GP passes my address on to this official)
(4) I would like to answer this question on a form **on the internet**
(5) I feel that no one may ask me this question, if I would be diagnosed with an STI

Below you find the questions that you would be asked, if you would be diagnosed with an STI.
Who would you allow to ask the following questions? You can choose more than one possibility for each question.

	My GP	someone else (on the phone)	someone else (by mail)	I answer the question on an internet form	no one is allowed to ask me this question
Which symptoms do you have?	☐	☐	☐	☐	☐
Did you have an STI before?	☐	☐	☐	☐	☐
Do you have an idea who might have passed the infection on to you?	☐	☐	☐	☐	☐
Do you have a regular sex partner?	☐	☐	☐	☐	☐
Do you have occasional sex partners?	☐	☐	☐	☐	☐
When might you have gotten the infection?	☐	☐	☐	☐	☐
Where (in which town) might you have gotten the infection?	☐	☐	☐	☐	☐
Did you visit a prostitute?	☐	☐	☐	☐	☐

Part two:some information about you

1. Gender: ☐ man ☐ woman

2. Age:...................years

3. In which country were you born?.............................

4. In which country was your mother born?....................

5. In which country was your father born?.......................

6. What is the highest qualification you have (or are getting now)?

Primary school	☐	Professional education	☐
Lower secondary school	☐	College (short type)	☐
General higher secondary school	☐	College (long type)	☐
Technical higher secondary school	☐	University	☐

7. Do you have one regular GP? ☐ Yes ☐ No

8. Is your GP a ☐ man ☐ woman

9. How often do you visit a GP? ....times a year

10. What is the zip code of your home address?..................

11. Do you use the internet? ☐ Yes ☐ No

12. Have you ever had a sexually transmitted infection (STI)? ☐ Yes ☐ No

13. Have you ever had yourself tested for an STI (including an HIV-test ? ☐ Yes ☐ No

14. How many sex partners have you had in the past year?

None	☐	4-10	☐
1	☐	>10	☐
2-3	☐		

15. Do you have (or have you ever had) sexual contact with

A man ☐
A woman ☐
Both ☐

16. If you would have an STI, your sex partner(s) should get a treatment too.

I would inform my partner(s) myself ☐
I would allow my GP to inform my partner(s) ☐
I would allow someone else, who is specially appointed for this purpose, to inform my partner(s) ☐
No one is allowed to inform my partner(s) ☐

The end – thank you!!
